# Machine Learning Electrocardiogram for Mobile Cardiac Pattern Extraction

**DOI:** 10.3390/s23125723

**Published:** 2023-06-19

**Authors:** Qingxue Zhang, Dian Zhou

**Affiliations:** 1 Department of Electrical and Computer Engineering, Department of Biomedical Engineering, Purdue School of Engineering and Technology, 723 W. Michigan St., Indianapolis, IN 46202, USA; 2 Department of Electrical and Computer Engineering, University of Texas at Dallas, 800 W Campbell Rd, Richardson, TX 75080, USA; zhoud@utdallas.edu

**Keywords:** smart health, ECG, machine learning, pattern recognition, medical decision support

## Abstract

Background: Internet-of-things technologies are reshaping healthcare applications. We take a special interest in long-term, out-of-clinic, electrocardiogram (ECG)-based heart health management and propose a machine learning framework to extract crucial patterns from noisy mobile ECG signals. Methods: A three-stage hybrid machine learning framework is proposed for estimating heart-disease-related ECG QRS duration. First, raw heartbeats are recognized from the mobile ECG using a support vector machine (SVM). Then, the QRS boundaries are located using a novel pattern recognition approach, multiview dynamic time warping (MV-DTW). To enhance robustness with motion artifacts in the signal, the MV-DTW path distance is also used to quantize heartbeat-specific distortion conditions. Finally, a regression model is trained to transform the mobile ECG QRS duration into the commonly used standard chest ECG QRS durations. Results: With the proposed framework, the performance of ECG QRS duration estimation is very encouraging, and the correlation coefficient, mean error/standard deviation, mean absolute error, and root mean absolute error are 91.2%, 0.4 ± 2.6, 1.7, and 2.6 ms, respectively, compared with the traditional chest ECG-based measurements. Conclusions: Promising experimental results are demonstrated to indicate the effectiveness of the framework. This study will greatly advance machine-learning-enabled ECG data mining towards smart medical decision support.

## 1. Introduction

Heart failure tops the list as the leading cause of death globally [1]. To manage heart diseases, for many decades, electrocardiograms (ECGs) have been used as a gold-standard vital signal, which intrinsically encode complex cardiac physiological processes [2]. However, traditional in-clinic/hospital ECG-based heart health management is usually expensive and inconvenient, which is a major impediment either for real-time cardiac emergency prediction or for long-term chronic heart disease tracking.

Nowadays, advancement in internet-of-things technologies, such as mobile data capturing and signal processing/machine learning techniques [3,4], are enabling more and more out-of-clinic daily health applications [5,6]. Many promising practices in health management, COVID management, mobile health, and data-driven precision medicine have been advanced [7,8]. Many studies have also been reported in terms of ECG-based heart health monitoring. A chest-worn ECG device is the most popular solution [9,10], but it usually suffers from uncomfortableness/inconvenience due to the chest strap and sweating. Other studies place the ECG electrodes on both wrists or both index fingers for ECG acquisition [11,12], which usually cause inconvenience and discontinuity in monitoring.

Generally, it is highly desirable to explore convenient and comfortable health management systems to enable long-term continuous and real-time precision medicine [1,6,8,13,14,15,16,17,18,19,20,21]. We have previously reported a highly wearable ear-worn blood pressure monitoring system, in which the mobile ECG is used to determine the heartbeat occurrence time [22]. However, no further study has been carried out to explore other potential uses of the highly subtle mobile ECG, which is acquired using a nonstandard but unobstructive lead configuration that can provide superior convenience and comfort. It will greatly advance long-term use because a nonstandard ECG lead configuration does not require the strict placement of electrodes on the two arms or the chest under the cloth. However, this also poses a great challenge to analyze the signals that are sensitive to noise and/or interference. We name this kind of nonstandard and highly convenient lead configuration “mobile ECG” to distinguish it from traditional limb- or chest-based configurations. Mobile ECG is very suitable for long-term use, compared with many other studies’ configurations, which usually use the interlimb ECG that requires a wire or the touch of a finger, or the chest ECG that may need a chest strap [1,6,8,13,14,15,16,17,18,19,20,21]. In this study, we take special interest in whether the mobile ECG can be used to track the duration of the QRS complex, which is the central part of an ECG heartbeat. QRS duration carries a great deal of medical information and has been reported to be relevant to many heart diseases, such as a coronary disease that may cause sudden death [23], a right ventricular disease that reduces blood volume [24], and many other diseases [25,26,27,28,29].

**Figure 1:**
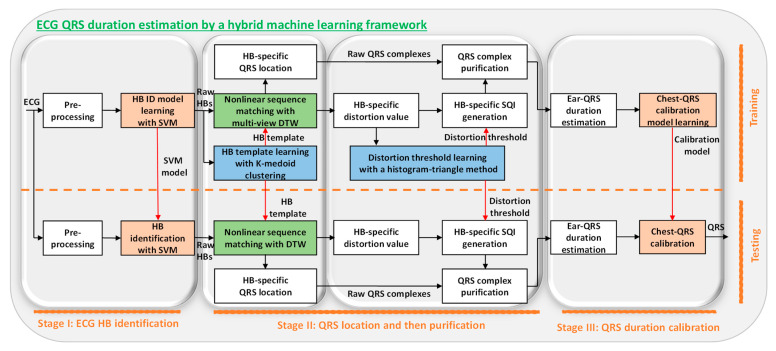
System diagram of novel machine learning framework, which identifies the heartbeats with the support vector machine (SVM) in stage I, localizes the QRS complexes with the multiview dynamic time warping (MV-DTW) in the first part of stage II, purifies the QRS complexes in the second part of stage II, and then transforms the mobile-ECG-based QRS durations to commonly used standard chest-ECG-based ones in stage III. The blocks above the dashed line are for training, and those under the line are for testing, so the HB ID model, HB template learning, and distortion threshold learning are only included in the training phase. Notes: Orange blocks: supervised learning steps; blue blocks: unsupervised learning steps; green blocks: pattern recognition steps; HB: heartbeat; ID: identification; SVM: support vector machine; DTW: dynamic time warping.

Specifically, in this study, we propose a convenient mobile-ECG-based cardiac health management system for long-term real-time ECG QRS duration tracking. To the best of our knowledge, it is the first study on mobile-ECG-based QRS duration estimation for heart health management. The proposed system is empowered by a novel machine learning framework. With this challenging mobile ECG lead configuration, the signal acquired is very tiny and sensitive to motion artifacts; therefore, it is processed by the proposed sophisticated three-stage machine learning framework (Figure 1). Firstly, a support vector machine (SVM) [30] is introduced to identify raw heartbeats from the subtle mobile ECG. Afterwards, a multiview dynamic time warping (DTW) approach [11] is developed, not only to locate the raw QRS complex in each raw heartbeat, but also to quantize the quality of that heartbeat, referring to a predefined high-quality ECG heartbeat template that is learned using a K-medoid clustering method [31]. The raw heartbeat quality information is used to purify the raw QRS complexes by comparing with a quality threshold learned using a histogram triangle method [32]. Finally, the mobile ECG QRS duration is estimated and then a regression-based calibration model is learned to transform the mobile ECG QRS duration estimate to the commonly used standard chest ECG QRS duration. Promising experimental results are demonstrated to indicate the effectiveness of the proposed framework. This study will greatly advance machine-learning-enabled ECG data mining towards smart medical decision support.

Our contributions are summarized as below:

(1) The novel machine learning framework can intelligently and systematically identify the heartbeats from a noisy and subtle mobile ECG, localize the ECG QRS complexes, purify the complexes, and transform the mobile ECG QRS durations to the standard chest ECG QRS durations.
(2) The support vector machine classifier determines the raw heartbeats from the signal spikes, which include both real and false heartbeats that are due to the motion artifacts.
(3) The ECG QRS localization step leverages the multiview dynamic time warping for sophisticated pattern matching, to compare a given raw heartbeat with the high-quality heartbeat template determined using the k-medoid clustering method.
(4) The purification step further leverages the pattern matching scores to generate the signal quality indices and boost the performance.
(5) The transformation of mobile ECG QRS durations to the commonly used standard chest ECG QRS durations facilitates the convenient usage of the extracted cardiac pattern.

We will then detail the proposed novel machine learning algorithm, give the results and discussions, and conclude this study.

## 2. Materials and Methods

In this section, details of the proposed system are introduced according to the signal processing flow (Figure 1).

### 2.1. System Overview

The proposed system is shown in Figure 1, illustrating a three-stage framework leveraging advanced machine learning techniques for QRS duration estimation.

### 2.2. Stage I: ECG Heartbeat Identification

The filtered mobile ECG was learned by an SVM classifier [30] for raw heartbeat identification purposes as shown in Figure 1. Firstly, the ECG stream was segmented using an adaptive threshold approach to select signal spikes as the heartbeat candidate. Afterwards, ten critical motion-artifact-tolerant features were extracted [22], which were then fed into the SVM classifier to learn a heartbeat identification model. A supervised learning strategy was used to train the SVM classifier, with the chest ECG heartbeats as ground truths to differentiate real and false mobile ECG heartbeats in the learning process. The trained SVM decision model is as (1), where $x_{i}$/$y_{i}$/$\alpha_{i}$ are the i-th support vector/its class label/its learned weight factor, b is the learned bias, k is the kernel (a linear one is chosen to lower the computation load), x is a ten-dimension feature vector of a heartbeat candidate, and $f\left(x\right)$ is the predicted label (a raw heartbeat or a false heartbeat) [33].

(1) $$f\left(x\right)=sign(\sum_{i=1}^{V}\alpha_{i}y_{i}\cdot k\left(x,x_{i}\right)+b)$$

### 2.3. Stage II: QRS Localization and then Purification

After identifying raw heartbeats, we developed a pattern recognition approach, multiview dynamic time warping (DTW) [11], not only to locate raw QRS complexes, but also to quantize the raw heartbeat quality for purification purposes (due to motion artifacts, the raw heartbeats include many distorted heartbeats, which are to be shown in the Section 3).

Before the following processing steps, all heartbeats were segmented based on the heartbeat locations identified using the SVM and were scaled to be between 0 and 1. One thing worth noting is that these heartbeat segments are bounded by two adjacent R peaks, meaning that an ECG heartbeat segment mentioned in stage II and III of our framework actually includes the second half of a heartbeat and the first half of the following heartbeat. This segmentation method is based on the consideration that it will facilitate the determination of appropriate boundaries for highly subtle and noisy mobile ECG heartbeats by leveraging the most distinguishable R peaks as the natural heartbeat boundaries.

#### 2.3.1. Representative Heartbeat Template Learning by K-Medoid Clustering

DTW performed nonlinear sequence-to-template matching to determine the relation between a testing signal stream and a predefined template signal stream. To select a high-quality representative heartbeat as the template, we applied a k-medoid clustering method [31]. This approach leverages an unsupervised learning strategy to avoid manual template selection that is both inconvenient and subject to non-optimal selection.

Specifically, for each subject, we clustered all raw ECG heartbeats in the training session into K groups and selected the medoid as the template from the group that had the highest number of instances. K was set as 3, and the Euclidean distance was selected as the distance metric to lower the computation load (all heartbeats were resampled to possess the same length). Firstly, the initial medoid seeds were chosen using a K-means++ method [34] as in (2)–(3), where the j-th seed $c_{j}$ was selected from a set of resampled raw heartbeats $\hat{\Theta }$ with probability $w_{\hat{\theta_{j}}}$, which is proportional to the Euclidean distance between $\hat{\theta_{j}}$ and its closest preselected medoid $c_{p}$. $D_{p}$ is a set of all raw heartbeats closest to medoid $c_{p}$. Afterwards, the K-medoid problem was solved by a partitioning around medoids (PAMs) strategy [35] as in (4), which greedily checks if swapping each medoid $c_{j}$ and each nonmedoid c reduces the summarized instance-to-medoid dissimilarity ξ, until no progress can be obtained. Finally, a high-quality heartbeat representing most of the raw heartbeats was learned and selected as the DTW template as in (5). This learning process was performed for each subject, using the training data in the training phase.

(2) $$c_{j}=Select(\hat{\theta_{j}}|w_{\hat{\theta_{j}}},\forall \hat{\theta_{j}}\epsilon \hat{\Theta })\;$$

(3) $$w_{\hat{\theta_{j}}}=\frac{Euclidean\left(\hat{\theta_{j}},c_{p}\right)}{\sum_{\left\{h|\hat{\theta_{h}}\epsilon D_{p}\right\}}Euclidean\left(\hat{\theta_{h}},c_{p}\right)},\;\forall \hat{\theta_{j}}\epsilon \hat{\Theta },\;p<j$$

(4) $$\xi =\sum_{\left\{c_{p}|p=1,\ldots ,K\right\}}\sum_{\left\{h|\hat{\theta_{h}}\epsilon D_{p}\right\}}Euclidean\left(\hat{\theta_{h}},c_{p}\right)$$

(5) $$T=\underset{\forall c_{j}}{\mathrm{argmax}}\;Num(D_{j})$$

#### 2.3.2. QRS Localization by Multiview Dynamic Time Warping

To locate the QRS complex, the multiview DTW (MV-DTW) [11] was developed to nonlinearly match each raw heartbeat with the learned representative heartbeat. To reveal more signal characteristics, in addition to the original ECG amplitude series, another two dimensions (the first derivative series and the local angle series) were also extracted, constructing a three-view heartbeat representation which is more robust to motion artifacts, compared to the original single-view time series. To generate the angle dimension, for each sample, we calculated an angle defined by the current sample and its two neighbors (preceding and following the current sample) with a distance of 10 samples to capture the piece-wise fluctuations.

The testing heartbeat $\theta_{j}$ of a length of M and the template T of a length of N (both have three dimensions) are shown in (6)–(10), respectively, where j is the heartbeat index. MV-DTW-based nonlinear stream matching includes three steps. Firstly, a local distance matrix is generated to evaluate all sample-to-sample distance possibilities, with each element $d^{m,n}$ defined as (10). Secondly, a path distance table is constructed by a dynamic programming strategy as (11), where each element $D^{m,n}$ is the summation of current local distance $d^{m,n}$ and the minimum of three preceding neighboring path distance elements. Thirdly, the QRS onset $QRS_{j}^{on}$ and offset $QRS_{j}^{off}$ in the testing heartbeat $\theta_{j}$ are located using a backward search method as shown in (12) and (13), not only referring to the QRS boundaries ($QRS_{Temp}^{on}$ and $QRS_{Temp}^{off}$) in the template T, but also according to the optimal warping path information learned when generating the path distance table D. Leveraging MV-DTW and the learned high-quality heartbeat template, the QRS boundaries are expected to be automatically located, from the highly weak and noisy mobile ECG heartbeats.

(6) $$\theta_{j}=\left\{\theta_{j,1},\theta_{j,2},\theta_{j,3}\right\}$$

(7) $$T=\left\{T_{1},T_{2},T_{3}\right\}$$

(8) $$\theta_{j,l}=\left\{\theta_{j,l}^{m}|0\leq m\leq M-1\right\},\;\forall l$$

(9) $$T_{l}=\{T_{l}^{n}|0\leq n\leq N-1\},\;\forall l$$

(10) $$d_{j}^{m,n}=\sqrt{\sum_{l=0}^{L-1}{\left(\theta_{j,l}^{m}-T_{l}^{n}\right)}^{2}}\;\;\;\;\forall m,\;\forall n,\;L=3\;$$

(11) $$D_{j}^{m,n}=\left\{\begin{array}{cc} d_{j}^{m,n}+\min\left\{\begin{array}{c} D_{j}^{m-1,n}\\ D_{j}^{m,n}\;\;\;\;\\ D_{j}^{m,n-1} \end{array}\right. & \forall m>0\;\&\;\forall n>0\\ d_{j}^{m,n} & m=0\;\&\;n=0\\ inf & otherwise \end{array}\right.\;$$

(12) $$QRS_{j}^{on}=BackSearch\left(QRS_{Temp}^{on},\;D_{j}\right)$$

(13) $$QRS_{j}^{off}=BackSearch\left(QRS_{Temp}^{off},\;D_{j}\right)$$

#### 2.3.3. Heartbeat Distortion Quantization

The mobile ECG is highly weak and sensitive to motion artifacts, resulting from the highly challenging nonstandard single-lead configuration (more visualization will be shown in the Results section to illustrate distorted QRS complexes). Therefore, to further enhance the robustness, we propose quantizing the quality of raw heartbeats identified by the SVM classifier and purifying the raw QRS complexes located by the MV-DTW. The raw heartbeat distortion $\pi_{j}$ is defined by (14), which corresponds to the stream-to-stream path distance $D_{j}^{m,n}$ calculated by the MV-DTW. (14) is a special case of (11), by setting *m* = *M* and *n* = *N*. A large distance results in a high distortion value, and vice versa.

(14) $$\pi_{j}=D_{j}^{M,N}$$

#### 2.3.4. Distortion Threshold Learning by Histogram Triangle Search

To perform binary quality labelling (high- or low-quality) for raw heartbeats, a distortion threshold is learned from raw heartbeat distortion values using a histogram-triangle search method [32], for each subject (training data). A left-skewed histogram can be generated based on the heartbeat distortion values, leveraging the fact that high-quality heartbeats usually concentrate in the left part of the histogram (low distortion values) and low-quality raw heartbeats (distorted and false heartbeats) usually spread over a large range due to highly diverse behaviors caused by motion artifacts.

A global search method was applied to find the threshold τ that corresponds to a transition point in the histogram, which possesses the maximum perpendicular distance to the histogram hypotenuse as defined in (15), where b is a bin in the histogram, $h\left\{b\right\}$ is a function returning the density value for bin b, and Dis{} is a function returning the distance from a point $(\left(b,h\left(b\right)\right)$ to the hypotenuse that is drawn between the maximum point $(\left(b_{max},h\left(b_{max}\right)\right)$ and the rightest point $(\left(b_{right},h\left(b_{right}\right)\right)$. The learned distortion threshold can reflect the natural histogram transition point between high and low-quality raw heartbeat. An unsupervised learning strategy was used here since manual threshold selection is both inconvenient and subject to nonoptimal selection.

(15) $$\tau =\underset{b_{\max}\leq b\leq b_{right}}{\mathrm{argmax}}Dis\left\{(\left(b,h\left(b\right)\right),h\left(b_{max}\right)\sim h\left(b_{right}\right)\right\}$$

#### 2.3.5. QRS Purification

Leveraging quantized raw heartbeat distortion conditions and the learned distortion threshold, we could then generate raw heartbeat-specific signal quality indices (SQIs) to filter out low-quality raw QRS complexes. To further enhance the robustness, besides the calculated distortion sequence $\pi_{j}$, a smoothed version $\eta_{j}$ was also generated as (16) by a moving average operation with an order of 10 (A = 10). Both sequences were compared with the distortion threshold and the index $SQI_{j}$ for the i-th raw heartbeat was finally defined by (17).

(16) $$\eta_{j}=\sum_{a=0}^{A-1}\pi_{j-a}/A,\;\forall j$$

(17) $$SQI_{j}=\left\{\begin{array}{cc} 1 & \mathrm{If}\;\pi_{j}\leq \tau \;\mathrm{and}\;\eta_{j}\leq \tau \\ 0 & otherwise \end{array}\right.\;,\;\forall j$$

### 2.4. Stage III: QRS Duration Calibration

#### 2.4.1. QRS Duration Estimation

After purifying the raw ECG QRS complexes, we could obtain heartbeat-specific QRS duration estimates based on their boundaries. To improve estimation accuracy, we further averaged the estimates over each one-minute datum of interest in each trail. Therefore, we had fifteen averaged mobile ECG QRS estimates for both training and testing sessions for each subject.

#### 2.4.2. Mobile QRS Duration to Chest QRS Duration Calibration

Considering that QRS durations estimated using different lead configurations (such as lead II-wrists and lead IV-chest) may also be different [36], a bias parameter was learned to transform the mobile ECG QRS duration estimates to the chest-ECG-based ones, for each subject using the training data. The bias parameter was calculated based on the average QRS duration with the mobile ECG, and the average QRS duration with the chest EST, meaning that their difference was used as an adjusting factor for the QRS duration with the mobile ECG. More complex calibration models (nonlinear or higher-order) were not considered, not only to prevent an overfitted model but also to fairly reflect how mobile-ECG-based estimates can mimic chest-ECG-based ones.

## 3. Results and Discussion

In this section, we give detailed experimental results and a discussion according to the signal processing flow in Figure 1. Except for some learning steps, we use all testing data to visualize the results to take into account the generalization ability of the algorithm.

### 3.1. Experimental Setup

**Figure 2:**
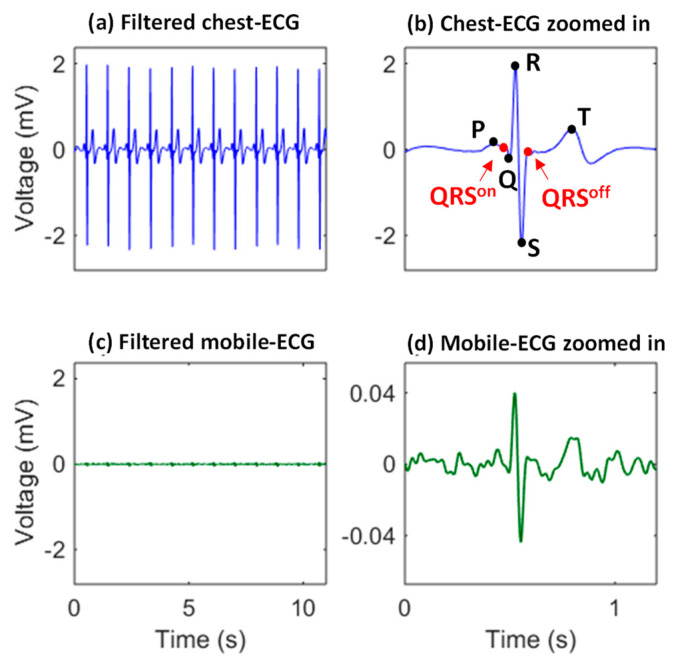
The mobile ECG (**c**) owns distinguishable morphologies, but is much weaker and more sensitive to motion artifacts, compared with the traditional chest-ECG (**a**). P, R, R, S, and T, are characteristic points of an ECG heartbeat. The on and off positions of the QRS complex are also labeled in the chest-ECG zoomed in (**b**), and the corresponding mobile ECG zoomed in is also given (**d**) to demonstrate the mobile ECG is of a very low bio-potential and sensitive to noise.

The mobile ECG dataset with the ECG signal from the very convenient area (the ear [22]) was used. The subtle and motion-artifact-sensitive mobile ECG from eight subjects was preprocessed using a six-order Butterworth bandpass filter (2–30 Hz) for baseline wander suppression and power line interference removal. Each subject had two recordings as the training and testing, respectively, and each recording had thirty minutes. The raw and filtered signals are shown in Figure 2, indicating the low amplitude and the noisy characteristics of the mobile ECG which pose a great challenge for cardiac pattern extraction.

### 3.2. Heartbeat Identification

**Figure 3:**
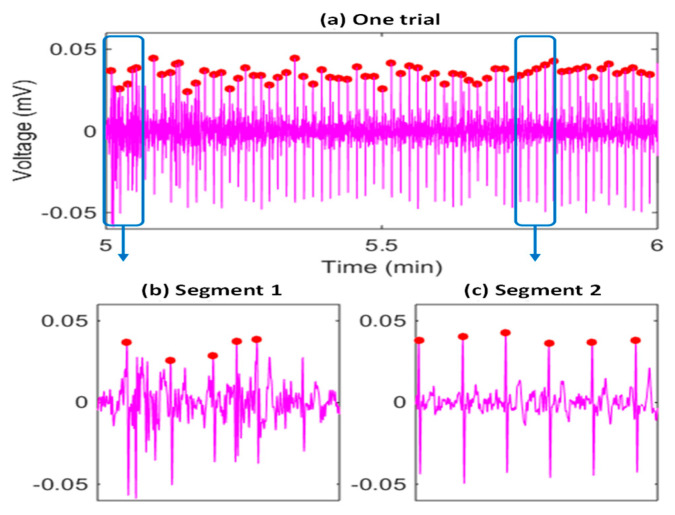
An example of the SVM-based heartbeat identification results (**a**), corresponding to the second minute data of one trial in the testing session of a subject (subject 6 is selected as an example), and showing the necessity to deal with many highly distorted heartbeats and some faking heartbeats. Two segments are further visualized with more details in (**b**,**c**). The red dots are detected heartbeat R peak locations.

The heartbeat locations are determined in the first stage of the proposed framework, which are used to segment the raw heartbeats that will be used in the following QRS location and purification steps. An example of the heartbeat identification results is given in Figure 3, where the red dots correspond to the identified mobile ECG heartbeats, indicating most of the mobile ECG heartbeats were successfully located (e.g., the segment 2 in Figure 3c).

At the same time, we can observe that during some segments (e.g., segment 1 in Figure 3b), there may be many highly distorted heartbeats and some false heartbeats, due to the weakness of the signal, exercise stress, and head movements. Firstly, a nonstandard mobile ECG signal is highly weak, and the peak-to-peak voltage is only around 2% to 5% of that of a traditional chest ECG signal. Secondly, it is impractical to ask monitor wearers to stay strictly still, and thus, there are always some motion artifacts. Thirdly, we asked the participants to ride a bike to introduce exercise stress and more variability to the heartbeat morphologies/intervals. Fourthly, to make the system suited to real-world application scenarios, we further deliberately introduced twenty-second head movements in the second minute (the duration used in algorithm evaluation) of each trail. All the above reasons usually result in low-quality raw heartbeats (distorted or false ones), and significantly impact the QRS duration estimation. Therefore, we propose purifying the raw heartbeats to improve the robustness of the ear-worn system.

### 3.3. Representative Heartbeat Learned by Clustering

**Figure 4:**
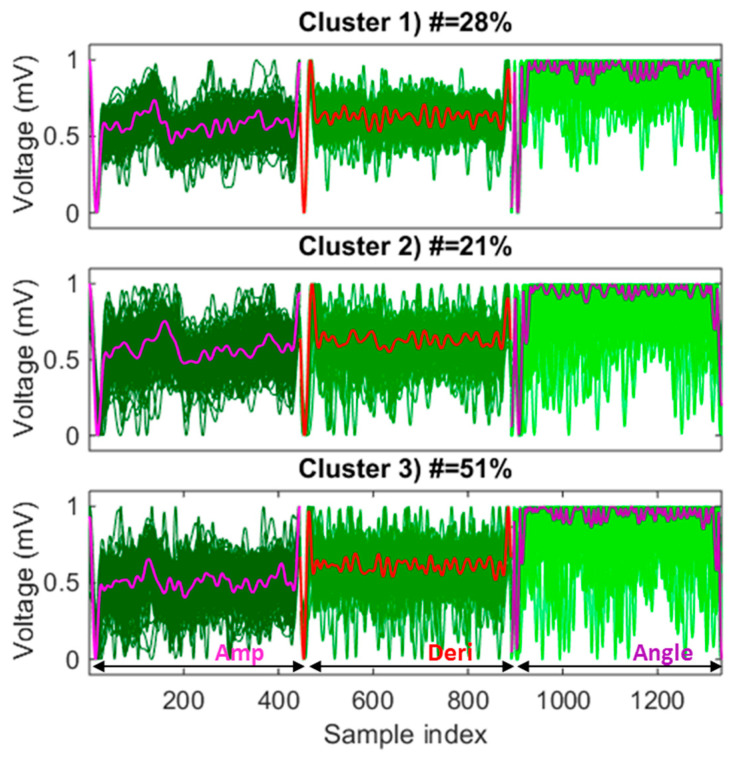
An example of a representative heartbeat learned by k-medoid clustering, corresponding to the training session of subject 6, where the medoid in cluster 3 is selected as a high-quality template since it represents the highest number of raw heartbeats (51%) among three medoids. Dark to blue lines: different dimensions of the three-dimensional raw heartbeats; bold lines: different dimensions of the medoids; all dimensions in all raw heartbeats are scaled to be between 0 and 1. Notes: Amp—amplitude; Deri—derivative.

A representative heartbeat was learned with the k-medoid clustering method, which will be used as a template by the MV-DTW approach, for both QRS location and purification purposes. Figure 4 shows an example of the learning results, where three-dimensional raw heartbeats are grouped into three clusters to differentiate their behaviors. The medoid that represents the highest number of raw heartbeats was selected as the representative heartbeat, i.e., the medoid in cluster 3 in this example. One thing worth noting is that three dimensions of each heartbeat were concatenated and fed into the k-medoid algorithm to more effectively visualize each dimension. Figure 4 also shows that the raw ECG heartbeat actually possesses a very low signal-to-noise ratio and is, thus, highly sensitive to noise and motion artifacts. However, the k-medoid clustering approach successfully learned a three-dimensional representative heartbeat with much better morphologies compared with other raw heartbeats.

### 3.4. QRS Located by Multiview DTW

**Figure 5:**
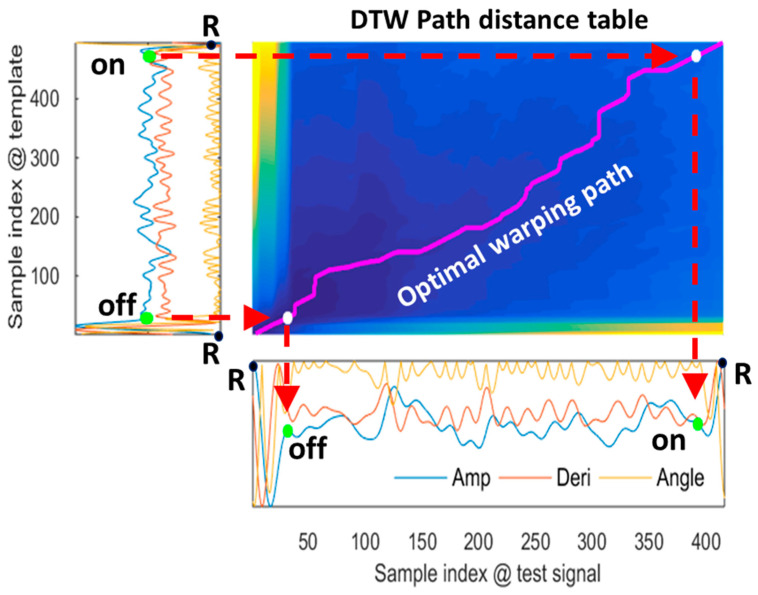
An example of the multiview-DTW-based nonlinear stream matching, corresponding to a raw heartbeat in the testing session of subject 6, and showing that the QRS offset/onset in the three-dimensional test stream is robustly located using a backward search method referring to the locations in the template stream. To determine the off and on boundaries of the QRS complex of the mobile ECG in the bottom, the corresponding preknown off and on locations in the chest ECG template on the left side are firstly mapped to the optimal warping path in the middle graph and then mapped to the mobile ECG in the bottom. Notes: the yellow/blue colors correspond to the highest/lowest path distance values, respectively. Amp—amplitude; Deri—derivative.

MV-DTW was applied to nonlinearly match a heartbeat with the learned representative high-quality heartbeat in order to determine point-to-point relations between them, as shown in Figure 5. Optimal warping path encoding nonlinear matching results were generated in the path distance table using a dynamic programming strategy. The ORS boundaries (the offset of one QRS complex and the onset of the following QRS complex, in an R-peak-to-R-peak heartbeat) in the test heartbeat were robustly located using a backward search method referring to the boundary locations in the template. This multiview DTW will be shown to be more tolerant to motion artifacts than the traditional single-view DTW in the next performance summary, leveraging amplitude/derivative/angle information which reveals meaningful point/pair/piece-wise signal characteristics.

### 3.5. Distortion Quantization and Threshold Learning

**Figure 6:**
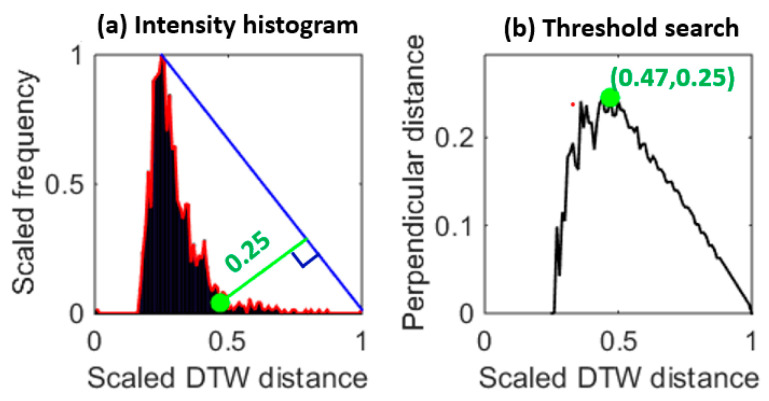
An example of the histogram triangle-based distortion threshold learning (**a**), corresponding to the training session of subject 6, and showing that the transition point (greed dot on the left side) is learned to determine an appropriate distortion threshold (0.25 with normalization). The right plot (**b**) is based on the distance from each point on the right side of the red envelop to the blue line in the left plot, so each distance is actually corresponding to a perpendicular line to the blue line. The green dot in the right plot is maximum, meaning that there is a sharp transition in the red envelop in the left plot and this transition is a good threshold.

MV-DTW also generates a stream-to-stream path distance, which can be used to quantize the quality condition of a raw heartbeat. With these heartbeat-specific distortion values, a threshold is needed to label these heartbeats as high- or low-quality ones. The threshold learning process is shown in Figure 6. The left-skewed intensity histogram of MV-DTW distances (Figure 6a) indicates the fact that high-quality heartbeats usually concentrate in a low-distortion area, while low-quality ones (distorted or false heartbeats) spread over a larger range due to the diversity induced by motion artifacts. Therefore, the transition point in Figure 6a can be determined using the global search method shown in Figure 6b, and the corresponding DTW distance was, thus, used as a distortion threshold.

### 3.6. Heartbeat Quality Labelling and Purification

**Figure 7:**
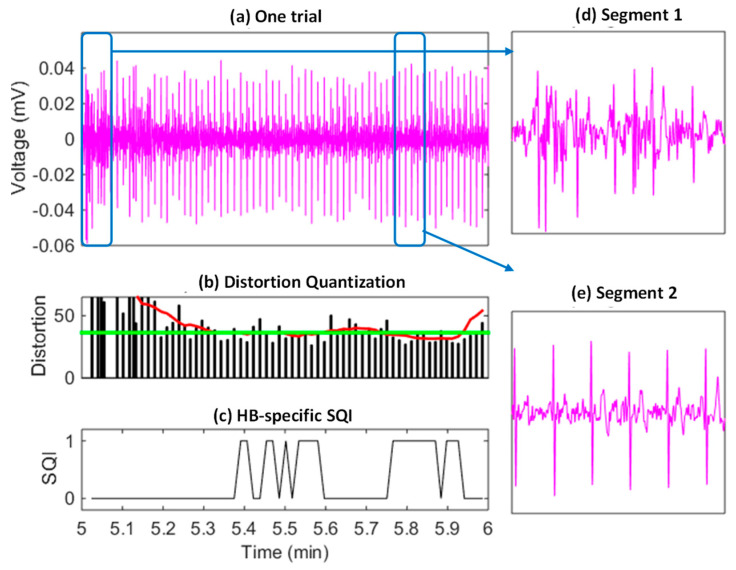
An example of the SQI-based raw heartbeat purification (**a**), corresponding to the testing session of subject 6, and indicating that segments highly distorted by motion artifacts have been successfully filtered out and other segments with a high quality are reserved. Red/green curves in (**b**): the smoothed distortion sequence and the distortion threshold, respectively. Two segments are further visualized to demonstrate the high-noise condition (**d**) and relative the normal-noise condition (**e**).

Based on the heartbeat distortion values and the learned threshold, the heartbeat-specific SQIs were generated as shown in Figure 7, where (a), (b), and (c) correspond to a one-minute mobile ECG segment, heartbeat-specific distortion values, and SQIs, respectively. The time-varying heartbeat-specific quality conditions can be effectively reflected by the distortion values generated by MV-DTW, which were used to generate the SQIs for heartbeat purification. In such a way, the ECG segments with severe motion artifacts (e.g., the segment 1 in Figure 7d) can be filtered out and the high-quality segments (e.g., the segment 2 in Figure 7e) are reserved. One thing worth noting is that the purification is relatively strict to filter out suspicious raw heartbeats and may result in much fewer remaining heartbeats. The scarification of heartbeats with moderate-quality conditions is meaningful, such that the impact from heartbeats can be effectively suppressed to a large degree, which will be demonstrated in the next performance summary.

### 3.7. Mobile QRS to Chest QRS Calibration

**Figure 8:**
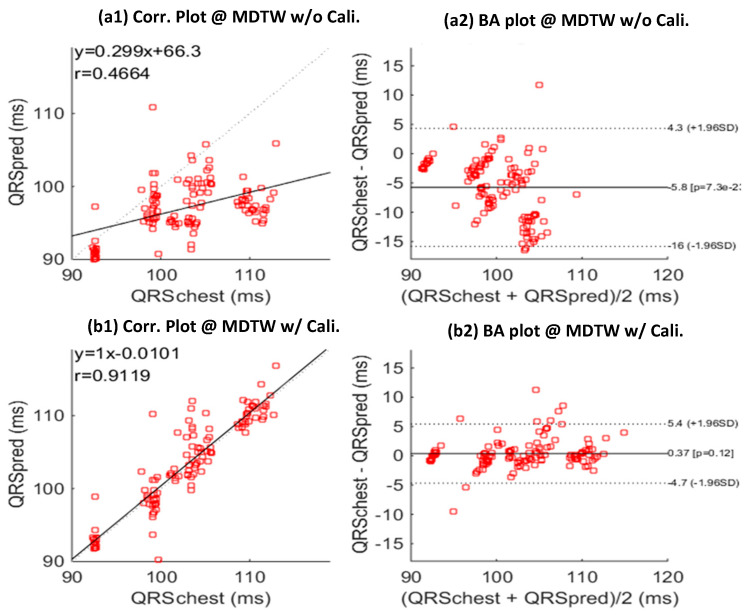
Correlation (Corr.) and Bland-Altman (BA) plots between predicted chest-QRS durations (QRSpred) and real chest-QRS durations (QRSchest), without (**a**,**b**) calibration (Cali.) model learning. Results is based on the testing sessions of all subjects; MDTW: MV-DTW. (**a1**) and (**b1**) give the correlation plots, and (**a2**,**b2**) give the BA plots.

Due to the QRS duration difference between different lead configurations mentioned before, we introduced a calibration model which was learned based on the training data of each subject. This model can effectively calibrate the mobile ECG QRS duration estimates to predict the standard chest ECG QRS duration estimates, as shown in Figure 8, where the prediction results without and with calibration are visualized in Figure 8(a1,a2,b1,b2), respectively. With calibration, the correlation efficient was improved by 91.2% to 46.6% (without calibration), and the distribution in the BA plot was significantly improved, resulting in a smaller mean error and also a much lower standard deviation.

### 3.8. Performance Summary

The mobile-ECG-QRS-based chest QRS prediction performance is summarized in Table 1, where the last row corresponds to the proposed framework, and other rows give some simpler options for comparison purposes. There are several interesting observations which can be made. Firstly, approaches with MV-DTW (rows 5–8) showed much better performance compared with those with DTW (rows 1–4), indicating that the MV-DTW can effectively capture more nonlinear patterns in sequence matching and distortion quantization. Secondly, SQI-based purification can effectively contribute to performance improvement, showing that the strict purification operation successfully suppressed most of the unreliable QRS duration estimates due to distorted and false raw heartbeats. Thirdly, the model calibration method was also significant in compensating the ear-lead-to chest-lead QRS duration bias, resulting in a further performance enhancement. With the proposed framework that has MV-DTW, SQI-based purification, and model calibration as shown in Figure 1, the prediction performance is very encouraging, and the correlation coefficient, mean error ± standard deviation, mean absolute error, and root mean absolute error are 91.2%, 0.4 ± 2.6, 1.7, and 2.6 ms, respectively.

### 3.9. Future Studies

It will be promising to further enhance the system with more effective pattern extraction [37,38,39,40] studies. The proposed approaches could also be generalized to other applications or signals [41,42,43,44] for event detection, template signal learning, and signal quality purification. These promising possibilities will deepen the current research and broaden its impact in the era of big medical data.

## 4. Conclusions

In this study, focusing on one major impediment faced by pervasive out-of-clinic ECG-based heart health tracking, i.e., the low level of convenience, we designed and validated a highly convenient cardiac management system by leveraging a novel machine learning framework for noisy mobile ECG signal analysis. We took a special interest in ECG QRS duration tracking, which carries a large amount of medical information and relates to many heart diseases [23,24,25,26,27,28,29]. Firstly, raw heartbeat locations were identified by an SVM classifier. Secondly, QRS boundaries were located with a novel MV-DTW approach, referring to a high-quality heartbeat template learned by a k-medoid clustering method. At the same time, the MV-DTW path distance was used to quantize the distortion conditions of raw heartbeats, which were then compared with a distortion threshold learned by a histogram triangle method to generate heartbeat-specific signal quality indices for purification purposes. Finally, the estimated mobile ECG QRS durations were transformed to the commonly used standard chest ECG QRS durations. Promising experimental results were demonstrated, indicating the effectiveness of the proposed framework. This study will greatly advance machine-learning-enabled ECG data mining towards smart medical decision support.

## Figures and Tables

**Table 1:** Ear-QRS-based Chest QRS Estimation Performance Summary on Testing Sessions of All Subjects.

APPROACHES	CR	ME	STD	MAE	RMSE
DTW	−2.1%	10.9	23.8	15.3	26.1
DTW + SQI	−4.9%	8.0	24.8	16.4	26.0
DTW + Cal.	48.5%	2.2	8.2	5.8	8.4
DTW + SQI + Cal.	54.1%	2.7	7.8	5.1	8.2
MV-DTW	28.3%	−3.6	6.2	5.4	7.1
MV-DTW + SQI	46.6%	−5.8	5.1	6.2	7.7
MV-DTW + Cal.	84.1%	−0.3	3.4	2.3	3.4
MV-DTW + SQI + Cal. (Proposed)	91.2%	0.4	2.6	1.7	2.6

Notes. Cal.: model calibration; CR: correlation efficient; ME: mean error; STD: standard deviation; MAE: mean absolute error; RMSE: root mean square error; unit of error: ms.

## Data Availability

Not applicable.
